# Cytotoxic Effects of 5-Azacytidine on Primary Tumour Cells and Cancer Stem Cells from Oral Squamous Cell Carcinoma: An In Vitro FTIRM Analysis

**DOI:** 10.3390/cells10082127

**Published:** 2021-08-19

**Authors:** Valentina Notarstefano, Alessia Belloni, Simona Sabbatini, Chiara Pro, Giulia Orilisi, Riccardo Monterubbianesi, Vincenzo Tosco, Hugh J. Byrne, Lisa Vaccari, Elisabetta Giorgini

**Affiliations:** 1 Department of Life and Environmental Sciences, Università Politecnica Delle Marche, 60131 Ancona, Italy; v.notarstefano@univpm.it (V.N.); a.belloni@pm.univpm.it (A.B.); c.pro@pm.univpm.it (C.P.); 2 Department of Material, Environmental Sciences and Urban Planning, Università Politecnica Delle Marche, 60131 Ancona, Italy; s.sabbatini@univpm.it; 3 Department of Clinical Sciences and Stomatology, Università Politecnica Delle Marche, 60126 Ancona, Italy; g.orilisi@pm.univpm.it (G.O.); r.monterubbianesi@univpm.it (R.M.); vtosc.92@gmail.com (V.T.); 4 FOCAS Research Institute, Technological University Dublin, Dublin 8, Ireland; hugh.byrne@TUDublin.ie; 5 Elettra Sincrotrone Trieste, SISSI Beamline, 34149 Basovizza, Italy; lisa.vaccari@elettra.eu

**Keywords:** oral squamous cell carcinoma, OSCC primary cells, OSCC cancer stem cells, Fourier Transform InfraRed Microscospectroscopy, DNA methylation, 5-azacytidine

## Abstract

In the present study, the cytotoxic effects of 5-azacytidine on primary Oral Squamous Cell Carcinoma cells (OSCCs) from human biopsies, and on Cancer Stem Cells (CSCs) from the same samples, were investigated by an in vitro Fourier Transform InfraRed Microscospectroscopy (FTIRM) approach coupled with multivariate analysis. OSCC is an aggressive tumoral lesion of the epithelium, accounting for ~90% of all oral cancers. It is usually diagnosed in advanced stages, and this causes a poor prognosis with low success rates of surgical, as well as radiation and chemotherapy treatments. OSCC is frequently characterised by recurrence after chemotherapy and by the development of a refractoriness to some employed drugs, which is probably ascribable to the presence of CSCs niches, responsible for cancer growth, chemoresistance and metastasis. The spectral information from FTIRM was correlated with the outcomes of cytotoxicity tests and image-based cytometry, and specific spectral signatures attributable to 5-azacytidine treatment were identified, allowing us to hypothesise the demethylation of DNA and, hence, an increase in the transcriptional activity, together with a conformational transition of DNA, and a triggering of cell death by an apoptosis mechanism. Moreover, a different mechanism of action between OSSC and CSC cells was highlighted, probably due to possible differences between OSCCs and CSCs response.

## 1. Introduction

Oral Squamous Cells Carcinoma (OSCC), representing ~90% of all oral cancers, is an aggressive tumoral lesion of the epithelium, characterised by invasive growth and frequent lymph nodes metastasis; moreover, despite the easy access of oral cavity for clinical examination, it is usually diagnosed in advanced stages, which inevitably accounts for a poor prognosis and a marked detriment of life quality [1,2]. Despite the improvements in surgical techniques and radiation and chemotherapy treatments, OSCC incidence is still increasing, particularly in patients aged between 50 and 70 years, with the 5-year survival rate remaining unchanged at ~54% over the past 30 years [3,4]. In this light, the need of further insights into the OSCC response to chemotherapy treatments, together with the mechanisms of cancer initiation, progression and tumour recurrence, are crucial to discover new therapeutic targets and to develop more effective therapeutic strategies. In addition to the severity of the pathology, OSCC is also characterised by recurrence after chemotherapy and the development of a refractoriness to some employed drugs [5,6,7]. It is now clear that, in part, this chemoresistance can be assigned to the presence of cancer stem cells (CSCs) niches, displaying stem cells clonogenic properties, able to generate new tumours and to stimulate cancer growth, chemoresistance, and metastasis [8,9]. The chemotherapy treatment may trigger the onset of aggressive phenotypes, or the development of a sub-population of chemoresistant CSCs [10,11,12]. With these concepts in mind, the need to include CSCs in studies focused on providing new insights into the effects of chemotherapy drugs is crucial, for acquiring a comprehensive knowledge on the mechanism of response of the pathology, possibly improving the clinical outcome of treatments.

Nowadays, in addition to studies focused on tumour genetics, there is a growing interest and knowledge about the epigenetic modifications occurring in several types of cancer, including OSCC. DNA methylation is an epigenetic mechanism of interest in cancer research, consisting of the transfer and covalent binding of a methyl group to a cytosine in CpG dinucleotides by DNA methyltransferases (DNMTs); the methylation of CpG islands, accumulated in the 60% of 5′ promoter regions of genes, determines a transcriptional repression [9]. As regards OSCC onset and carcinogenesis in general, DNA hypermethylation represents a widely studied epigenetic alteration, linked to the methylation and silencing of important tumour suppressor genes [9,13]. In this light, in order to attempt the reactivation of the expression of genes silenced by DNA methylation, including tumour suppressor genes, several DNA demethylating agents have been designed and investigated, including 5-azacytidine and 5-aza-2′-deoxycytidine [14]. These demethylating agents are incorporated into the DNA strand and act as a natural substrate for DNMTs, which initiates the reaction of methylation, but becomes covalently bound to DNA, due to the presence of a nitrogen instead of the carbon-5: besides the block of the DNMT function, the formation of a DNA-protein adduct prevents normal DNA functionality and triggers DNA damage signalling, leading to its final degradation [15,16]. Moreover, 5-azacytidine may also act on active demethylation pathway, upregulating the expression of the TET2 enzyme and increasing the abundance of cytosine derivatives in hepatocancer cells and human fibroblasts [17,18]. There is also indication that the cytosine analogue 5-aza-2′-deoxycytidine acts on the active DNA demethylation pathway in HeLa cells [19].

Some phenotypes typical of CSCs, acquired by suppressing gene expression, are based on epigenetic reprogramming, which may, hence, be a potential target for therapy against the whole cellular population of the tumour, including tumoral cells and cancer stem cells [9,20].

Fourier Transform Infrared Microspectroscopy (FTIRM) is a vibrational spectroscopic technique, widely and successfully applied in life sciences to investigate the bio-molecular structure and composition of cells and tissues [21,22,23,24,25]. By FTIRM, it is possible to obtain, on the same sample and at the same time, information on the structure and chemical composition of the cellular components. Indeed, the analysis of IR band position, intensity, and width provides the molecular fingerprints of proteins, lipids, carbohydrates, and nucleic acids within the investigated samples. In two previous studies, FTIRM and Raman Microspectroscopy were exploited to assess the effects of cisplatin and 5-fluorouracil on primary tumoral cells from OSCC, highlighting a time-dependent drug-specific cellular response; the same approach implemented for CSCs evidenced the onset of a mechanism of chemoresistance and enrichment of resistant CSCs as a result of cisplatin treatment [26,27].

In the present study, we combined an in vitro FTIRM approach and multivariate analysis, to assess the cytotoxic effects of 5-azacytidine on primary OSCC cells from human biopsies, and on cancer stem cells, obtained with sphere formation from the same human OSCC samples. The spectral information from FTIRM was correlated with the outcomes of cytotoxicity tests and image-based cytometry, with the aim of highlighting spectral signatures attributable to 5-azacytidine treatment and to assess possible differences between OSCCs and CSCs response.

## 2. Materials and Methods

The study, approved by the Ethics Committee of Azienda Ospedaliero Universitaria–Ospedali Riuniti Ancona, was carried out in full accordance with ethical principles for experiments involving humans, including The Code of Ethics of the World Medical Association (Declaration of Helsinki). 

Patients were subjected to a standard surgical procedure with diagnostic purposes and signed an informed consent to participate to this investigation; moreover, for privacy rights, a code was assigned to each sample, to anonymise the patients.

### 2.1. Culture of Primary OSCC Cells

For obtaining primary OSCC cells (hereafter OSCCs), a procedure previously validated was adopted [26,27]. Briefly, oral biopsy samples from lesions with diagnosis of poorly differentiated OSCC (tumour grade 3, assessed by histological analysis) were collected from 5 patients (3 males and 2 females; mean age 51.0 ± 2.5 years). All biopsy samples were carefully chopped into small fragments and cultured at 37 °C in a humidified atmosphere with 5% CO_2_, in Dulbecco’s modified Eagle’s medium (DMEM F-12), supplemented with 10% foetal bovine serum (FBS) and 1% penicillin–streptomycin solution. The growth medium was changed every 24 h and cell overgrowth was controlled by selective trypsinisation (0.05% trypsin–0.02% EDTA); cells were routinely tested for mycoplasma contamination. Once 90% of confluence was reached, primary OSCC cells were detached with trypsin, counted by a haemocytometer, centrifuged (1200 rpm for 5 min), and pooled together.

### 2.2. Culture of OSCC Cancer Stem Cells

For the sphere formation assay and cancer stem cells (hereafter CSCs) enrichment, the protocol described in [27] was followed. Briefly, OSCC cells were plated at a density of 1 × 10^5^ cells/well in 6-well ultra-low attachment plates in DMEM F-12 medium, supplemented with 2% FBS, human recombinant epidermal growth factor (hrEGF, 10 ng/mL), and fibroblast growth factor-basic (bFGF, 20 ng/mL). Fibroblasts spread after two weeks, and after one month a different cell population appeared, characterised as epithelial by immunocytochemical analysis with a cytokeratin antibody. During CSCs-enrichment, the culture medium was changed every 24 h, and fresh aliquots of hrEGF and bFGF were supplemented every 2 days, until the formation of tumour spheres [28].

### 2.3. Cell Viability Assay

The 5-azacytidine (Merck KGaA, Darmstadt, Germany) concentration that reduced the viability of OSCCs and CSCs by 50% (IC_50_) was determined by means of the colorimetric MTT (3-(4,5-dimethylthiazol 2yl)diphenyltetrazolium bromide) assay [29]. Cellular viability was evaluated by the ability of mitochondria to reduce the yellow MTT reagent to a purple formazan product. OSCCs and CSCs were seeded in 96-well plates (10 × 10^3^ cells per well) in DMEM F-12, respectively, with 10% FBS and 2% FBS, 20 ng/mL hrEGF and 10 ng/mL bFGF; cells were incubated at 37 °C in a humidified atmosphere with 5% CO_2_. After 24 h, increasing concentrations of 5-azacytidine (0.1, 0.2, 0.4, 0.8, 1.0 μM for OSCCs and 0.1, 0.5, 0.4, 1.0, 1.5, 2.0 μM for CSCs) were added and cells were incubated for further 24 h at 37 °C and in a humidified atmosphere with 5% CO_2_ [26]. The experiments were repeated in triplicate. Both OSCCs and CSCs were compared with the respective control. Cells were then incubated with 5 mg/mL of MTT for 3 h at 37 °C; at the end of the treatment, the medium containing MTT was removed and 100 μL of dimethylsulphoxide (Merck KGaA, Darmstadt, Germany) were added to each well. The number of viable cells was correlated to the intensity of the purple formazan product, measured at 570 nm, by means of a microplate reader (Synergy HT, Biotek, Winooski, VT, USA). The ratios between the absorbance values (Abs) measured for drug-treated cells (named OSCC-5Aza and CSC-5Aza) and those of untreated ones (named OSCC-Ctrl and CSC-Ctrl) were calculated and reported in viability curves, as percentages of viable cells against the inhibitory effect of the treatments on the cellular mitochondrial activity: (Abs_OSCC-5Aza_/Abs_OSCC-Ctrl_) × 100 and (Abs_CSC-5Aza_/Abs_CSC-Ctrl_) × 100. The maximum of cellular metabolic activity (100%) was assumed for untreated control samples. The drug concentration that reduced the viability of cells by 50% (IC_50_) was determined by applying a linear regression between triplicate cellular data points and the 5-azacytidine concentration range (OriginPro 2018b software, OriginLab Corporation, Northampton, MA, USA).

### 2.4. Apoptosis Assessment by Annexin V/PI Staining

The apoptotic index of OSCCs and CSCs after treatment with 5-azacytidine, was evaluated by the Tali™ Apoptosis assay kit-Annexin V Alexa Fluor^®^ 488 and propidium iodide (Invitrogen, Milan, Italy). OSCCs were seeded into 6-well plates (2 × 10^5^ cells per well) in 3 mL DMEM F-12 with 10% FBS and incubated overnight at 37 °C in a humidified atmosphere with 5% CO_2_. Then, 5-azacytidine, at the IC_50_ calculated concentration (0.8 μM), was added and incubated for 24 and 48 h at 37 °C in a humidified atmosphere with 5% CO_2_. Similarly, CSCs were seeded into 6-well plates (2 × 10^5^ cells per well) in 3 mL DMEM F-12 with 2% FBS, 20 ng/mL hrEGF, 10 ng/mL bFGF, and incubated overnight at 37 °C in a humidified atmosphere with 5% CO_2_. Then, 5-azacytidine, at the IC_50_ calculated concentration (1.5 μM), was added and incubated for 24 and 48 h at 37 °C in a humidified atmosphere with 5% CO_2_. For both OSCCs and CSCs, additional cell aliquots were normally cultured without any chemotherapy treatment for 24 and 48 h. All the experiments were carried out in triplicate for a total of *n* = 24. For each time range, all cell samples were washed twice with ice-cold PBS (pH 7.4), centrifuged, resuspended in 100 μL of binding buffer (BB) (10^6^ cell/mL) and incubated with 5 μL of Tali^TM^ Annexin V Alexa Fluor^®^ 488 for 20 min at room temperature, in the dark. Cells were then centrifuged at 1000 rpm for 5 min, resuspended in 100 μL of BB and 1 μL of Tali^TM^ Propidium Iodide (PI) for an additional 5 min at room temperature in the dark and analysed with flow cytometer (Tali^®^ image-based cytometer). After staining, absent or low fluorescence was indicative of live cells, green fluorescence (positive to Annexin V and negative to PI) of early apoptotic cells, and red/green (positive to both dyes) fluorescence of late apoptotic cells [26]. The percentages of live (L), early apoptotic (EA), and late apoptotic/dead (LA/D) cells were determined based on the respective fluorescence histograms compared with control ones [30].

### 2.5. OSCCs and CSCs In Vitro Treatment for FTIRM Measurements

OSCCs and CSCs were seeded in 6-well plates (2 × 10^5^ cells per well) in DMEM F-12 and incubated at 37 °C in a humidified atmosphere with 5% CO_2_ with different formulations (10% FBS for OSCCs and 2% FBS, 20 ng/mL hrEGF and 10 ng/mL bFGF for CSCs). After 24 h, all cellular samples were treated with 5-azacytidine (calculated IC_50_: 0.8 μM for OSCCs, and 1.5 μM for CSCs) for 24 and 48 h (OSCC-5Aza-24, OSCC-5Aza-48, CSC-5Aza-24, and CSC-5Aza-48 experimental groups). Other aliquots of OSCCs and CSCs were normally cultured with no chemotherapy drug for 24 and 48 h (OSCC-Ctrl-24, OSCC-Ctrl-48, CSC-Ctrl-24, CSC-Ctrl-48 experimental groups). The study was not performed beyond the time point of 48 h, due to the achievement of the maximum confluence and the consequent cell detachment.

At each selected time point, the culture medium was collected, OSCCs and CSCs were harvested using 0.5% trypsin solution with 0.2% EDTA and centrifuged at 1200 rpm for 5 min. To eliminate FBS residues, pellets were washed twice with 100 µL DMEM F-12, centrifuged again at 1200 rpm for 5 min and fixed in 4% paraformaldehyde (PFA) for 15 min; after fixation, cells were washed twice in physiological solution, and then stored at 4 °C until FTIRM measurement.

### 2.6. FTIRM Measurements and Data Analysis

FTIRM measurements were performed at the Chemical and Life Sciences branch of the Infrared Beamline SISSI (Synchrotron Infrared Source for Spectroscopic and Imaging), Elettra Sincrotrone Trieste (Trieste, Italy) (Proposal N. 20155263). A Hyperion 3000 Vis-IR microscope coupled with a Vertex 70V interferometer and equipped with a HgCdTe (MCT_A) detector and (Bruker Optics, Ettlingen, Germany) was used.

OSCCs and CSCs were resuspended with 15 μL of NaCl and placed, without any further treatment, into a specific, in-house built, biocompatible IR transparent microfluidic device for in vitro FTIRM measurements. The device consists of two CaF_2_ optical windows (0.5 mm thick, 13 and 10 mm diameter, respectively), spaced apart 7.5 μm [31].

For each sample, ~60 areas (30 × 30 μm^2^) of densely packed cell monolayers were selected by visible microscopy and the corresponding IR spectra were collected in transmission mode in the MIR region (4000–800 cm^−1^), averaging 512 scans (spectral resolution 4 cm^−1^, zero-filling factor 2, scanner velocity 40 kHz). Background, acquired on a 1 mm CaF_2_ clean window, and buffer medium spectra were collected using the same parameters. All the collected spectra were corrected for the contribution of atmospheric carbon dioxide and water vapor with the atmospheric compensation routine of OPUS 7.5 software (Bruker Optics, Ettlingen, Germany); the spectral contributions of the medium were subtracted by running an in-house optimised Matlab routine [26,31]. Hence, IR spectra displaying a peak height at 1660 cm^−1^ (Amide I band of proteins) lower than 0.07 a.u. were discarded. The remaining preprocessed spectra were vector-normalised and converted in second derivative mode (Savitzky–Golay filter, 9 points of smoothing) (OPUS 7.5 software, Bruker Optics, Ettlingen, Germany).

Preprocessed spectra were then subjected to multivariate analysis, with no further preprocessing. Principal components analysis (PCA) was employed as an unsupervised multivariate approach to analyse spectral data of OSCCs and CSCs (OriginPro 2018b software, OriginLab Corporation, Northampton, MA, USA) [32]. The PCA of OSCC-Ctrl-24/OSCC-Ctrl-48/CSC-Ctrl-24/CSC-Ctrl-48 spectral data was performed to compare the spectral profiles of OSCCs and CSCs populations, with no treatment; in addition, the pairwise PCA between OSCC-Ctrl-24/OSCC-5Aza-24, OSCC-Ctrl-48/OSCC-5Aza-48, CSC-Ctrl-24/CSC-5Aza-24, and CSC-Ctrl-48/CSC-5Aza-48 spectral data were performed to evaluate the spectral modifications induced by the treatment at each time point. All the PCA comparisons were performed separately on the selected regions of interest (ROI) 3050–2800 cm^−1^ and 1300–900 cm^−1^. The 1780–1480 cm^−1^ spectral region, referred to as Amide I and II bands of proteins, was not taken into account, since they were inevitably influenced by the water subtraction procedure.

In order to highlight the spectral differences between OSCCs and CSCs populations and to assess the effects induced by the 5-azacytidine treatment, for each experimental group, the average absorbance spectrum and its standard deviation spectrum (average absorbance spectra ± standard deviation spectra) were calculated (averaging routine, OPUS 7.5 software). These spectra were curve fitted in the 3050–2800 cm^−1^ and 1300–900 cm^−1^ spectral regions, upon straight baseline correction and vector normalisation. The underlying bands were selected based on second derivative analysis and fixed before running the iterative process, to obtain the best reconstructed curve (residual close to zero; bandwidth 10 to 40 cm^−1^ range) (GRAMS/AI 9.1, Galactic Industries, Inc., Salem, NH, USA). For each underlying band, the integrated area was calculated and used for the following band area ratios: A_3010_/A_2925_, A_2925_/A_2960_, A_1171_/A_TOT_, A_1240_/A_1221_, A_1119_/A_1085_, A_1053_/A_TOT_, A_930_/A_970_, and A_915_/A_1085_. A_TOT_ was calculated by the sum of the integrated areas of all the underlying bands in the 1300 to 900 cm^−1^ spectral range. Significant differences between experimental groups were evaluated by means of a factorial analysis of variance (one-way ANOVA), followed by Tukey’s multiple comparisons test (software Prism6, Graphpad Software, Inc., San Diego, CA, USA). One-way ANOVA compares the means of OSCC-Ctrl-24, OSCC-Ctrl-48, CSC-Ctrl-24, CSC-Ctrl-48, OSCC-5Aza-24, OSCC-5Aza-48, CSC-5Aza-24, and CSC-5Aza-48 groups in order to make inferences as regards the population means. Statistical significance was set at *p* < 0.05.

### 2.7. Statistical Analysis

The IC_50_ values were determined by using a linear regression procedure (OriginPro 2018b software, OriginLab Corporation, Northampton, MA, USA). PCA was employed on spectral data as an unsupervised multivariate approach (OriginPro 2018b software); all the PCA comparisons were performed separately on the selected regions of interest (ROI) 3050–2800 cm^−1^ and 1300–900 cm^−1^. Normally distributed data derived from FTIRM spectra were presented as mean ± S.D; significant differences between experimental groups were determined by means of a factorial analysis of variance (one-way ANOVA), followed by Tukey’s multiple comparisons test, by the statistical software Prism6 (Graphpad Software, Inc., San Diego, CA, USA); One-way ANOVA compares the means of OSCC-Ctrl-24, OSCC-Ctrl-48, OSCC-5Aza-24, OSCC-5Aza-48, CSC-Ctrl-24, CSC-Ctrl-48, CSC-5Aza-24, and CSC-5Aza-48 groups in order to make inferences about the population means; statistical significance was set at *p* < 0.05; different letters over histograms indicate statistically significant differences among the above defined experimental groups.

## 3. Results

### 3.1. Dose-Response Curves and IC_50_ Values

**Figure 1:**
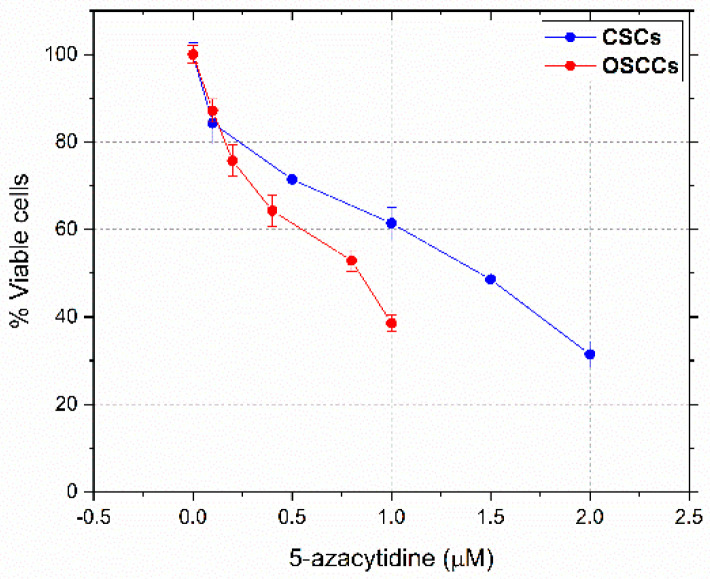
Dose-response curves for OSCCs treated with 5-azacytidine 0.1, 0.2, 0.4, 0.8, and 1.0 μM for 24 h (red line) and CSCs treated with 5-azacytidine 0.1, 0.5, 1.0, 1.5, and 2.0 μM for 24 h (blue line).

The MTT assay was performed to assess the sensitivity of OSCCs and CSCs towards 5-azacytidine. An in vitro dose-dependent anti-proliferative action of this drug, with respect to both OSCCs and CSCs, was highlighted by the viability curves shown in Figure 1. The calculated IC_50_ values (0.8 μM for OSCCs and 1.5 μM for CSCs) indicated that primary OSCCs are more sensitive to 5-azacytidine with respect to CSCs.

### 3.2. Apoptosis Rates

**Figure 2:**
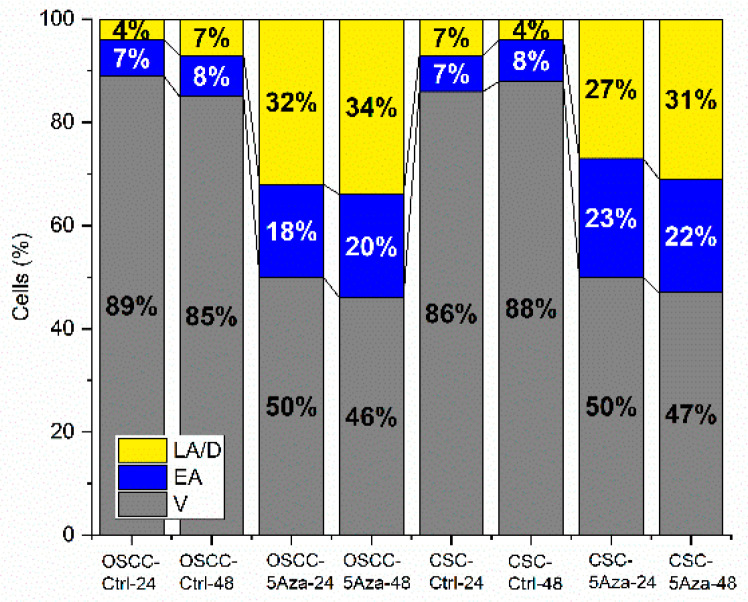
Percentages of viable (V), early apoptotic (EA) and late apoptotic/dead (LA/D) primary OSCC and CSC cells treated for 24 and 48 h with 0.8 and 1.5 μM 5-azacytidine, respectively (OSCC-5Aza and CSC-5Aza), and with no treatment (OSCC-Ctrl and CSC-Ctrl).

The percentages of viable (V), early apoptotic (EA) and late apoptotic/dead cells (LA/D) of OSCCs and CSCs populations, assessed after 24 and 48 h of treatment with 5-azacytidine (respectively, 0.8 and 1.5 μM), are reported in Figure 2. The results suggest that the 5-azacytidine treatment triggers cell death by means of apoptosis, with similar effects on both OSCCs and CSCs populations.

### 3.3. FTIRM Analysis

**Figure 3:**
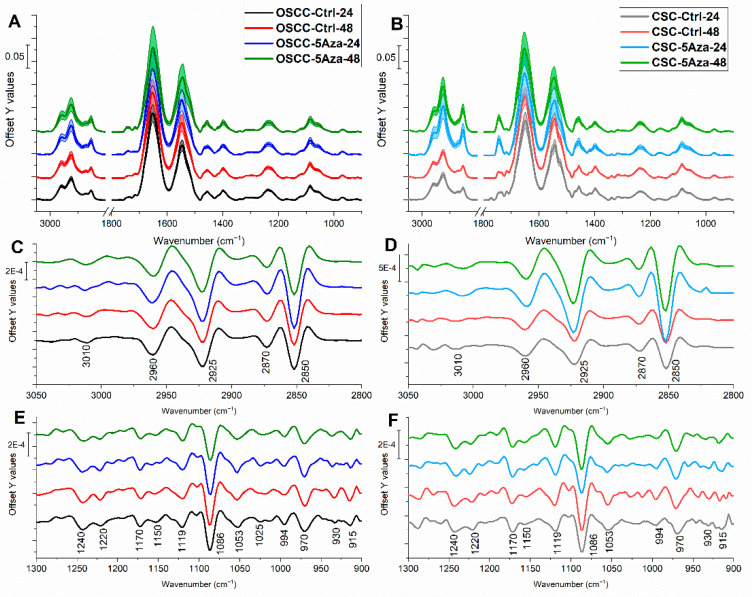
Average absorbance spectra in the 3050 to 2800 cm^−1^ and 1800 to 900 cm^−1^ spectral ranges of: (**A**) OSCC and (**B**) CSC cells. Second derivative spectra in the 3050 to 2800 cm^−1^ spectral range of OSCC (**C**) and CSC (**D**) cells. Second derivative spectra in the 1300 to 900 cm^−1^ spectral range of the OSCC cells (**E**) and CSC cells (**F**). For a better viewing, spectra are off set along y-axis. Labels along the second derivative spectra indicate the most relevant IR absorption bands.

The average IR spectra of all OSCCs and CSCs experimental groups were calculated, both in absorbance mode in the 3050 to 2800 cm^−1^ and 1800 to 900 cm^−1^ (Figure 3A,B) spectral ranges and in second derivative mode in the 3050 to 2800 cm^−1^ (Figure 3C,D) and 1300 to 900 cm^−1^ (Figure 3E,F) spectral ranges; labels along the second derivative spectra (Figure 3C–F) indicate the most relevant IR absorption bands, reported in Table 1 together with the related vibrational modes and biological assignments.

**Figure 4:**
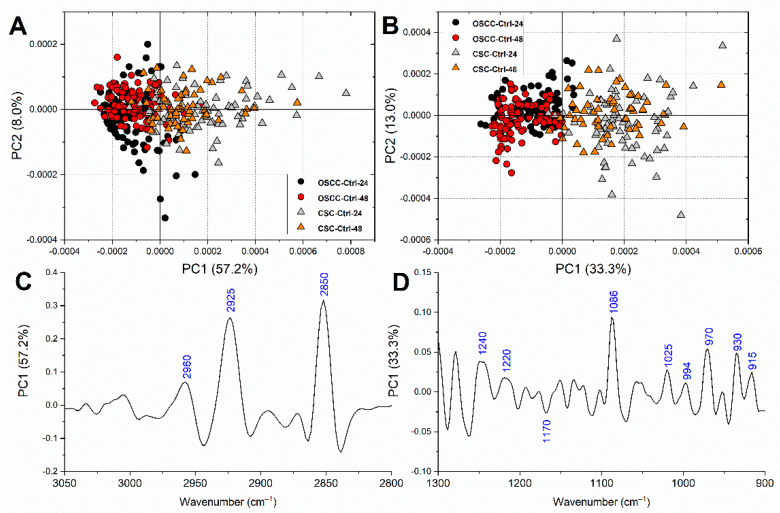
PCA scores plots calculated for OSCC-Ctrl-24, OSCC-Ctrl-48, CSC-Ctrl-24, and CSC-Ctrl-48 in the 3050–2800 cm^−1^ (**A**) and the 1300–900 cm^−1^ (**B**) spectral regions. Corresponding PC1 loading profiles in the 3050–2800 cm^−1^ (**C**) and the 1300–900 cm^−1^ (**D**) spectral regions. For clarity purposes, PCA loadings are plot with different Y scales for the two ROI (3050–2800 cm^−1^ and 1350–900 cm^−1^).

To highlight the differences between OSCCs and CSCs control groups, and to assess the homogeneity and stability within the two cell populations, PCA was first performed on all OSCC-Ctrl-24, OSCC-Ctrl-48, CSC-Ctrl-24, and CSC-Ctrl-48 spectral populations, both on the 3050–2800 cm^−1^ (lipids) and 1300–900 cm^−1^ (carbohydrates and nucleic acids) spectral regions. A clear segregation was found along PC1 between OSCCs and CSCs, while no separation was evidenced within OSCCs and CSCs populations at the two selected time points, both considering the regions related to lipids (Figure 4A) and carbohydrates/nucleic acids (Figure 4B). The PC1 loading on the lipid-related region showed the 2925 cm^−1^ and the 2850 cm^−1^, both assigned to CH_2_ groups of aliphatic chains, as the most discriminant spectral features (Figure 4C); as regards the PCA comparison performed on the nucleic acids-related spectral region, the most discriminant features were mostly related to nucleic acids and comprised the peaks centred at 1240 cm^−1^ (A-DNA), 1220 cm^−1^ (B-DNA), 1086 cm^−1^ (nucleic acids), 994 cm^−1^ (RNA), 970 cm^−1^ (DNA), 930 cm^−1^ (Z-DNA), and 915 cm^−1^ (RNA); moreover, the PC1 loading spectrum also evidenced the peaks related to carbohydrates and glycosylated compounds and centred at in 1025 cm^−1^ and 1171 cm^−1^ (Figure 4D).

**Figure 5:**
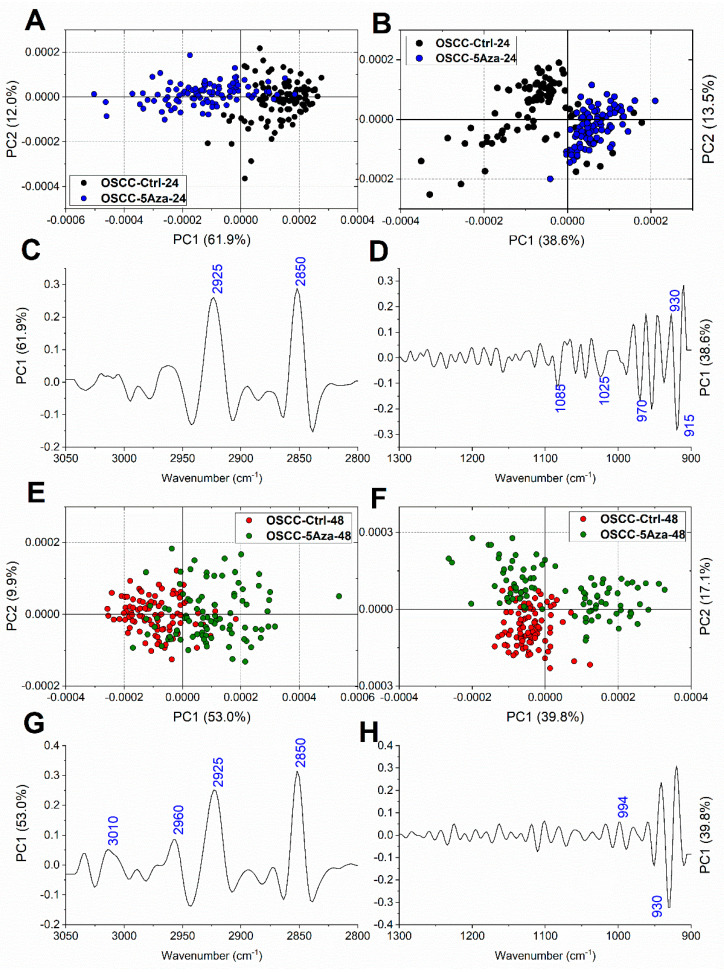
Pairwise PCA scores plots calculated for OSCC-Ctrl-24 and OSCC-5Aza-24 in the 3050–2800 cm^−1^ (**A**) and 1300–900 cm^−1^ (**B**) regions, and for OSCC-Ctrl-48 and OSCC-5Aza-48 in the 3050–2800 cm^−1^ (**E**) and the 1300–900 cm^−1^ (**F**) spectral regions. Corresponding PC1 loading profiles in the 3050–2800 cm^−1^ (**C**,**G**) and the 1300–900 cm^−1^ (**D**,**H**) spectral regions. For clarity purposes, PCA loadings are plot with different Y scales for the two ROI (3050–2800 cm^−1^ and 1350–900 cm^−1^).

Then, to precisely assess the spectral modifications induced by the 5-azacytidine treatment at each time point on both OSCCs and CSCs, the pairwise PCA comparisons OSCC-Ctrl-24/OSCC-5Aza-24, OSCC-Ctrl-48/OSCC-5Aza-48, CSC-Ctrl-24/CSC-5Aza-24, and CSC-Ctrl-48/CSC-5Aza-48 were performed, both in the lipid (3050–2800 cm^−1^) and nucleic acids (1300–900 cm^−1^) spectral regions. At 24 h of treatment, a good segregation between OSCC-Ctrl and OSCC-5Aza, both in the lipid and nucleic acids regions, was found (Figure 5A,B); in particular, the PC1 loadings evidenced a discrimination based on the 2925 cm^−1^ and 2850 cm^−1^ bands, both assigned to the CH_2_ groups of saturated lipid aliphatic chains (Figure 5C), and on bands related to nucleic acids, especially those centered at 970 cm^−1^ (DNA), 930 cm^−1^ (Z-DNA), and 915 cm^−1^ (RNA) (Figure 5D). Surprisingly, the PCA comparisons performed on OSCCs at 48 h displayed a different discrimination result; in fact, while a moderate segregation is visible in the lipid region (Figure 5E), the analysis performed on the nucleic acids regions evidenced the separation of 5-azacytidine-treated cells into two populations, one discriminated from the control cells along PC1 and one superimposed (Figure 5F). The PC1 loadings evidenced a contribution to the separation mostly relying on the lipids-related bands centered at 3010 cm^−1^ (=CH groups), 2960 cm^−1^ (CH_3_ groups), 2925 cm^−1^, and 2850 cm^−1^ (both CH_2_ groups) (Figure 5G), and on the 930 cm^−1^ band (Z-DNA) (Figure 5H).

**Figure 6:**
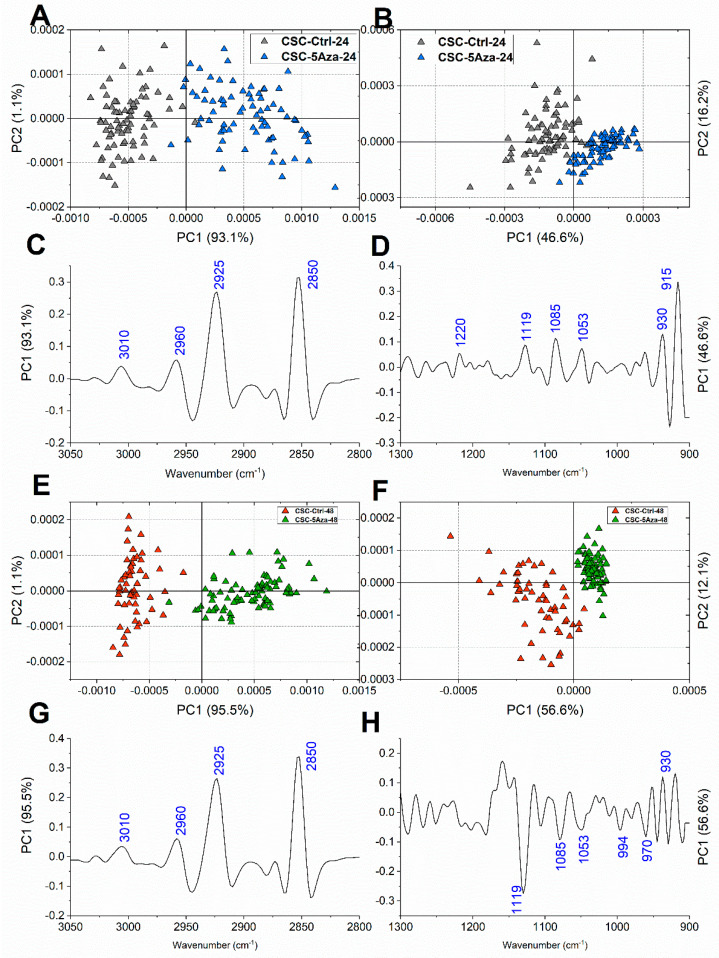
Pairwise PCA scores plots calculated for CSC-Ctrl-24 and CSC-5Aza-24 in the 3050–2800 cm^−1^ (**A**) and 1300–900 cm^−1^ (**B**) regions, and for CSC-Ctrl-48 and CSC-5Aza-48 in the 3050–2800 cm^−1^ (**E**) and the 1300–900 cm^−1^ (**F**) spectral regions. Corresponding PC1 loading profiles in the 3050–2800 cm^−1^ (**C**,**G**) and the 1300–900 cm^−1^ (**D**,**H**) spectral regions. For clarity purposes, PCA loadings are plot with different Y scales for the two ROI (3050–2800 cm^−1^ and 1350–900 cm^−1^).

As regards CSCs population, at 24 h of treatment, a good segregation was observed along PC1 between CSC-Ctrl and CSC-5Aza, both in the lipid and nucleic acids regions (Figure 6A,B, respectively); in particular, the PC1 loadings evidenced a discrimination based on the peaks centered at 3010 cm^−1^ (=CH groups), 2960 cm^−1^ (CH_3_ groups), 2925 cm^−1^, and 2850 cm^−1^ (CH_2_ groups) (Figure 6C), and on the bands related to nucleic acids centered at 1220 cm^−1^ (B-DNA), 1119 cm^−1^ (RNA), 1085 cm^−1^ (nucleic acids), 930 cm^−1^ (Z-DNA), and 915 cm^−1^ (RNA) (Figure 6D). The segregation along PC1 was also good after 48 h of treatment, with higher percentages of explained variance for both lipids and nucleic acids regions (respectively, Figure 6E,F); the analysis of the PC1 loadings confirmed the discriminant lipids-related peaks previously highlighted (Figure 6G), and evidenced for the nucleic acids region the major contribution of the peaks centered at 1119 cm^−1^ (RNA), 1085 cm^−1^ (nucleic acids), 970 cm^−1^ (DNA), and 930 cm^−1^ (Z-DNA) (Figure 6H).

**Figure 7:**
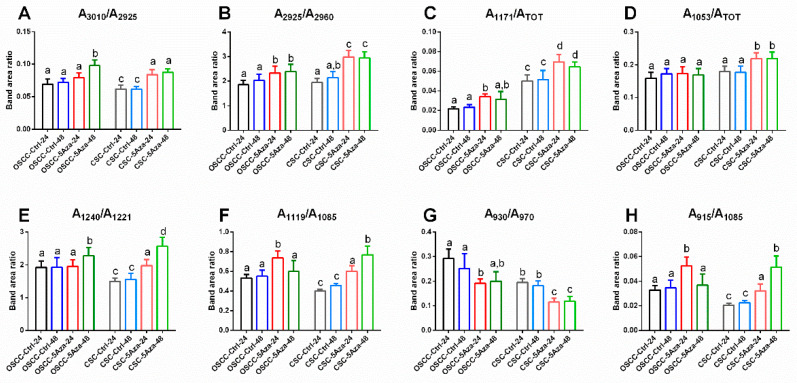
Histograms showing the numerical variation of the following band area ratios calculated for OSCC-Ctrl-24, OSCC-Ctrl-48, OSCC-5Aza-24, OSCC-5Aza-48, CSC-Ctrl-24, CSC-Ctrl-48, CSC-5Aza-24, and CSC-5Aza-48: (**A**) A_3010_/A_2925_, (**B**) A_2925_/A_2960_, (**C**) A_1171_/A_TOT_, (**D**) A_1053_/A_TOT_, (**E**) A_1240_/A_1221_, (**F**) A_1119_/A_1085_, (**G**) A_930_/A_970_, and (**H**) A_915_/A_1085_. Data are represented as mean ± SD. Different letters over histograms indicate statistically significant difference among groups (one-way ANOVA and Tukey’s multiple comparison test). Statistical significance was set at *p* <0.05.

For each experimental group, the average IR absorbance spectrum was calculated, together with its standard deviation spectra (average absorbance spectra ± standard deviation spectra), and several underlying bands with biological meaning were selected in the spectral ranges 3050 to 2800 cm^−1^ and 1300 to 900 cm^−1^ by curve fitting procedure, in order to interpret the information from loadings of second derivative spectra. The following band area ratios were then calculated and analysed, as follows (Figure 7): (i) in general, no statistically significant change was displayed in all the analysed band area ratios between OSCC-Ctrl-24 and OSCC-Ctrl-48, nor CSC-Ctrl-24 and CSC-Ctrl-48 groups, as suggested by the superimposition of spectra in the PCA scores plots in Figure 4A,B; (ii) the A_3010_/A_2925_ band area ratio, representing the degree of unsaturation of lipid aliphatic chains, displayed a significantly higher value respect to controls in all the treated groups, except for OSCC-5Aza-24 (Figure 7A); (iii) the A_2925_/A_2960_ band area ratio, representing the length/branching of lipid aliphatic chains, showed a significant increase in all the treated groups, with higher values displayed by CSC-5Aza respect OSCC-5Aza (Figure 7B); (iv) the A_1171_/A_TOT_ band area ratio, representing the C–C, C–O–C, and C–OH groups in carbohydrates, showed significantly higher values in general in CSCs respect to OSCCs; moreover, a significant increase induced by the 5-azacytidine treatment was found in both cell types, with the highest value at 24 h (Figure 7C); (v) the A_1053_/A_TOT_ band area ratio, representing the C–OH group in carbohydrates, displayed comparable levels among OSCC-Ctrl and CSC-Ctrl; furthermore, no significant change was induced by the 5-azacytidine treatment in OSCCs, while a significant increase in the ratio was found in CSCs (Figure 7D); (vi) the A_1240_/A_1221_ band area ratio, representing the amount of A-form DNA respect to B-form DNA, showed lower values in CSC-Ctrl respect to OSCC-Ctrl; moreover, the 5-azacytidine treatment induced a significant increase at 24 h in OSCCs (OSCC-5Aza-24) and at both times in CSCs /CSC-5Aza-24 and CSC-5Aza-48) (Figure 7E); (vii) the A_1119_/A_1085_ and A_915_/A_1085_ band area ratios, both representing the RNA cell content respect all nucleic acids, showed significantly higher values in treated groups, except for OSCC-5Aza-48, and displayed lower values in CSC-Ctrl respect to OSCC-Ctrl (Figure 7F,H); (viii) the A_930_/A_970_ band area ratio, representing the amount of Z-form DNA respect to the total double-stranded DNA, displayed significantly lower values in CSC-Ctrl respect to OSCC-Ctrl; moreover, in both cell types and at both times, the 5-azacytidine treatment induced a significant decrease in the ratio (Figure 7G).

## 4. Discussion

In the present study, the effects of 5-azacytidine on primary OSCC cells from human biopsies, and on cancer stem cells, obtained with sphere formation from the same human OSCC samples, were investigated in vitro by FTIRM spectroscopy. To the best of our knowledge, FTIRM has been recently applied on both these types of cells only to investigate the effects of routine chemotherapy drugs, such as cisplatin and 5-fuorouracil [26,27], but it was never exploited to analyse the role of 5-azacytidine, neither on immortalised cell lines nor on primary ones.

Oral Squamous Cells Carcinoma is an aggressive tumoral lesion of the epithelium and presents a 5-year survival rate of ~54%, partly due to the possibility of recurrence of the pathology after chemotherapy treatment and the onset of refractoriness to chemotherapy drugs [6,7]. For these reasons, the requirement of new insights on the mechanisms of OSCC response to chemotherapy is evident. In part, this chemoresistance is explained by the presence of Cancer Stem Cells niches, which are able to generate new tumours and to stimulate cancer growth and metastasis [8,9].

Since several types of cancer, including OSCC, are characterised by epigenetic alterations, such as modifications to DNA methylation of CpG islands, some chemotherapy drugs targeting these mechanisms have been designed and studied, such as 5-azacytidine [14].

In this work, the in vitro FTIRM analysis of primary OSCC cells and CSCs treated with 5-azacytidine was performed. To the best of our knowledge, although FTIR spectroscopy has been already used to assess the demethylation of valproic acid on the DNA of HeLa cells [48], this is the first time that this spectroscopic technique has been exploited to evaluate the effects of 5-azacytidine on primary nor immortalised cell lines.

The cellular viability and the apoptotic response of primary OSCCs and CSCs to these chemotherapy treatments were first evaluated by using MTT assay and image-based cytometry with AnnexinV/PI staining, respectively. Noteworthily, a higher effective dose was necessary for CSCs, indicating that primary OSCCs are more sensitive to 5-azacytidine in culture. Flow cytometry provided the percentages of viable, early apoptotic, and late apoptotic or dead cells of OSCCs and CSCs populations, assessed after 24 and 48 h of treatment with 5-azacytidine 0.8 and 1.5 μM, respectively: the obtained results suggested that the 5-azacytidine treatment triggers cell death by means of apoptosis, with similar effects on both OSCCs and CSCs populations. A slightly stronger effect was visible at 48 h of treatment, suggesting a time-dependent response. During FTIRM measurements, cells were maintained under controlled hydrated conditions in an in-house built biocompatible IR-transparent microfluidic device [31]. PCA first aimed to compare OSCCs and CSCs control groups, and to assess the homogeneity and stability within the two cell populations. The analysis evidenced a clear segregation of OSCCs and CSCs, suggesting a macromolecular difference between the two cell types, regarding both the lipid-related and nucleic acids-related spectral regions. Then, in order to precisely assess the spectral modifications induced by the 5-azacytidine treatment at each time point on both OSCCs and CSCs, the pairwise PCA comparisons OSCC-Ctrl-24/OSCC-5Aza-24, OSCC-Ctrl-48/OSCC-5Aza-48, CSC-Ctrl-24/CSC-5Aza-24, and CSC-Ctrl-48/CSC-5Aza-48 were performed. As regards OSCCs, at 24 h of treatment, a good segregation between OSCC-Ctrl and OSCC-5Aza was found in both the analysed spectral regions; surprisingly, the PCA comparisons performed on OSCCs at 48 h displayed a different discrimination result; in fact, while a moderate segregation was visible in the lipid region, the analysis performed on the nucleic acids regions evidenced the separation of 5-azacytidine-treated cells into two populations, one discriminated from the control cells along PC1 and one superimposed. This result may suggest the enrichment of a drug-resistant subpopulation and a reversion of the demethylating effects of 5-azacytidine on OSCCs. To the best of our knowledge, no information regarding the onset of a chemoresistance to 5-azacytidine in OSCC has been yet reported, however, it is known that the survival of tumoral cells rely on the DNA methylation of a few key regions, and these regions would be preferentially preserved from externally induced demethylation [14,49]. Conversely, the PCA comparisons performed on CSCs spectral data evidenced a clear segregation in both the selected spectral regions and after both 24 and 48 h of treatment. Hence, besides the known high heterogeneity of CSCs, often causing the enrichment of a drug-resistant cell subpopulation [27], 5-azacytidine homogeneously affects the entire CSCs population.

FTIRM data confirm, for both OSCCs and CSCs, a mode of action consistent with what is already reported in the literature. Briefly, 5-azacytidine acts by demethylating cellular DNA, with reactivation of silenced genes, and, by consequence of the chemical mechanism of its activity, it causes DNA damage due to the formation of irreparable, covalent protein-DNA adducts; all these effects, beyond a certain threshold, inevitably lead to cell death, in particular by apoptosis mechanisms [50]. To assess the demethylating effects of 5-azacytidine, the A_930_/A_970_ band area ratio, representing the amount of Z-form DNA respect to the total double-stranded DNA, was investigated [33]. Z-DNA is an elongated left-handed conformation of DNA, which can be found in segments with specialised sequences, characterised by alternations of deoxycytidine and deoxyguanosine residues, and the formation of which is facilitated by methylation [51,52]; the peculiar spatial conformation of Z-DNA can influence transcriptional activity by excluding transcription factors [53]. In both cell types, its values significantly decreased after 5-azacytidine treatment, confirming the demethylation activity of the drug; notably, the value of A_930_/A_970_ appeared significantly higher in OSCCs controls respect to CSCs ones, suggesting a different methylation state between the two cell populations. Since CpG islands, a major target of DNMTs, occupy 60% of promoter gene regions, their methylation is known to induce transcriptional repression. In this light, the amount of RNA in OSCCs and CSCs was also determined: as regards OSCCs, the A_1119_/A_1085_ and A_915_/A_1085_ band area ratios, both representing the RNA cell content with respect to all nucleic acids, showed significantly higher values only in OSCCs treated with 5-azacytidine for 24 h, while at 48 h their values were comparable to the control ones; on the contrary, CSCs cells showed a time-dependent response, with RNA values significantly increasing with respect to control over time. Notably, both the band area ratios displayed lower values in control CSCs with respect to OSCCs ones. The increase in RNA amount in cells treated with 5-azacytidine confirms the demethylation activity of the drug and the re-activation of epigenetically silenced promoter regions. Although FTIRM cannot provide information on which genes were re-activated by the demethylation of 5-azacytidine, the spectral data related to those of cell viability let suggest that some of the genes involved in tumour survival may have been the target of the drug. The formation of the irreparable, covalent DNMT-DNA adduct given by the 5-azacytidine incorporation into the DNA molecule determines a change in its conformation, until its break and degradation. To assess the degree of conformational change of DNA, the A_1240_/A_1221_ band area ratio, representing the amount of A-form DNA with respect to B-form DNA, was investigated. It is reported that the B- to A-DNA transition, besides occurring during dehydration, also arises during drug treatments, especially with compounds interrupting base pairing, determining the shift of DNA to more disordered A forms [27,37,44,47,54]. In OSCCs, the A_1240_/A_1221_ band area ratio significantly increased only after 48 h of treatment, while an increasing time-dependent trend was displayed by CSCs, again suggesting a different ongoing demethylating mechanism. It is known that apoptosis determines the accumulation of cytoplasmic lipid droplets: the onset of an apoptotic process may be suggested in 5-azacytidine-treated OSCCs and CSCs with an increase in the length and branching of lipid aliphatic chains (A_2925_/A_2960_) and an increase in unsaturated lipid aliphatic chains (A_3010_/A_2925_) [26,27,55], the latter also being a possible spectral marker of lipid oxidation [33,41,56]. Moreover, peculiar protein- phosphorylation events are known to occur during apoptosis, as evidenced by the significant increase in the A_1171_/A_TOT_ band area ratio in all treated cells [41,57]; additionally, this spectral feature appeared very different between OSCCs and CSCs: protein phosphorylation plays a crucial role in controlling cellular behaviour, affecting cellular growth, cell division, and metabolism; hence, the dysfunction in protein phosphorylation may contribute to the development and growth of cancers [58].

## 5. Conclusions

In this work, the cytotoxic effects of 5-azacytidine on primary tumour cells and cancer stem cells from OSCC biopsy samples were assessed by combining an in vitro FTIRM approach and multivariate analysis. The spectral information, correlated with the outcomes of MTT tests and image-based cytometry, let highlight specific spectral signatures attributable to 5-azacytidine treatment. In particular, the spectral results evidenced clear effects of the drug in demethylating DNA, re-activating transcription, inducing a conformational transition of DNA, and in triggering cell death by an apoptosis mechanism. FTIRM let also evidence a different response of OSCCs and CSCs to 5-azacytidine, which needs to be further investigated.

## Figures and Tables

**Table 1:** Centre position (wavenumber, cm^−1^), vibrational mode and biochemical assignment of the underlying bands identified by second derivative minima analysis of average and average ± S.D. absorbance spectra of OSCC-Ctrl-24, OSCC-Ctrl-48, OSCC-5Aza-24, OSCC-5Aza-48, CSC-Ctrl-24, CSC-Ctrl-48, CSC-5Aza-24, and CSC-5Aza-48 in the 3050 to 2800 cm^−1^ and 1300 to 900 cm^−1^ spectral range.

Wavenumber (cm^−1^)	Vibrational Mode and Biochemical Assignment
~3010	Stretching vibration of =CH moiety [33,34]
~2960,~2870	Antisymmetric and symmetric stretching vibrations of CH_3_ groups of branched aliphatic chains of lipids [27,35,36]
~2925, ~2850	Antisymmetric and symmetric stretching vibrations of CH_2_ groups of linear aliphatic chains of lipids [27,35,36]
~1240	Antisymmetric stretching vibrations of phosphate moieties of A-form DNA [27,37,38,39]
~1220	Antisymmetric stretching vibrations of phosphate moieties of B-form DNA [27,37,38,39]
~1170	Stretching vibration of non-hydrogen bonds of C-OP groups mainly in proteins [40,41]
~1119	Stretching vibration of the skeletal structure around the C2−OH group of RNA and NTPs [27,33,39,42]
~1086	Symmetric stretching vibrations of phosphate moieties in nucleic acids [26,27,43]
~1053	Stretching vibration of C-OH groups in carbohydrates and glycosylated compounds [27,35,42]
~1025	Stretching vibration of CH_2_-OH moieties in carbohydrates and glycosylated compounds [26,27,44]
~994	C–C, C–O ring breathing of RNA ribose [26,27,42,43]
~970	Backbone vibrations of nucleic acids, mainly double-strand DNA [25,42,43]
~930	Left-handed helix DNA vibrations (Z-DNA) [33,39,45,46,47]
~915	Ribose-phosphate skeletal vibrations in RNA [39,47]

## Data Availability

Data is contained within the article.
